# Utilization of the Caprini risk assessment model(RAM) to predict venous thromboembolism after primary hip and knee arthroplasty: an analysis of the Healthcare Cost and Utilization Project(HCUP)

**DOI:** 10.1186/s12959-024-00633-4

**Published:** 2024-07-24

**Authors:** Zhencan Lin, Hao Sun, Meiyi Chen, Deng Li, Zhiqing Cai, Yimin Wang, Jie Xu, Ruofan Ma

**Affiliations:** 1 Department of Orthopedics, Sun Yat-sen Memorial Hospital, Sun Yat-sen University, Guangzhou, 510120 Guangdong China; 2 Department of Orthopedics, The Eighth Affiliated Hospital, Sun Yat-Sen University, Shenzhen, 518000 Guangdong China; 3 School of Public Health, Sun Yat-sen University, Guangzhou, China

**Keywords:** Caprini risk assessment model, Total hip arthroplasty, Total knee arthroplasty, Venous thromboembolism, Healthcare Cost and utilization project (HCUP)

## Abstract

**Purpose:**

This study aims to investigate the potential role of Caprini risk assessment model (RAM) in predicting the risk of venous thromboembolism (VTE) in patients undergoing total hip or knee arthroplasty (THA/TKA). No national study has investigated the role of Caprini RAM after primary THA/TKA.

**Methods:**

Data from The National Sample of Healthcare Cost and Utilization Project (HCUP) in 2019 were utilized for this study. The dataset consisted of 229,134 patients who underwent primary THA/TKA. Deep vein thrombosis (DVT) and pulmonary embolism (PE) were considered as VTE. The incidence of thrombosis was calculated based on different Caprini scores, and the risk of the Caprini indicator for VTE events was evaluated using a forest plot.

**Results:**

The prevalence of VTE after primary THA/TKA in the U.S. population in 2019 was found to be 4.7 cases per 1000 patients. Age, body mass index (BMI), and Caprini score showed a positive association with the risk of VTE (*P* < 0.05). The receiver operating characteristic (ROC) curve analysis indicated that a Caprini score of 9.5 had a sensitivity of 47.2% and a specificity of 82.7%, with an area under the curve (AUC) of 0.693 (95% CI, 0.677−0.710). The highest Youden index was 0.299. Multivariate logistic regression analysis revealed that malignancy, varicose vein, positive blood test for thrombophilia, history of thrombosis, COPD, hip fracture, blood transfusion, and age were significant risk factors for VTE. Based on these findings, a new risk stratification system incorporating the Caprini score was proposed.

**Conclusions:**

Although the Caprini score does not seem to be a good predictive model for VTE after primary THA/TKA, new risk stratification for the Caprini score is proposed to increase its usefulness.

**Supplementary Information:**

The online version contains supplementary material available at 10.1186/s12959-024-00633-4.

## Introduction

With the aging of the population and the demand for a high quality of life, the incidence of joint replacement is increasing year by year. But for the postoperative complications of joint replacement, Venous thromboembolism (VTE), including deep vein thrombosis (DVT) and pulmonary embolism (PE), is a life-threatening complication of joint replacement [1]. In Europe and America, the incidence of DVT ranges from 2.22 to 3.29%, PE ranges from 0.87 to 1.99%, and fatal PE is 0.30% [2, 3]. In Asia, DVT is 1.4%, and PE is 1.1% [4]. In the US alone, VTE causes 100,000 to 180,000 deaths annually [5] and poses a significant burden on the healthcare system [6]. Major orthopedic surgeries are recognized to be at high risk for VTE, including hip fracture repair, total hip arthroplasty (THA), and knee arthroplasty (TKA) [7]. However, there are still many controversies in choosing preventive measures and assessing VTE risk. Both the American College of Chest Physicians (ACCP) and the American Association of Orthopaedic Surgeons (AAOS) agreed with the use of pharmacological prophylaxis for VTE after THA/TKA, but there is no consensus on the choice or dosage of the drug [8, 9]. Despite the routine use of anticoagulants or antiplatelets after surgery, VTE events still occur.

At present, the commonly used thrombosis risk assessment models are the Wells rule, Padua prediction score, and Caprini risk assessment model (Caprini RAM). However, there are questions about the validity of these models. One clinical trial indicated that the Wells score is more applicable to outpatients than inpatients [10] and Antonia Perez-Martin et al. reported the Wells score performed poorly for discrimination of risk for proximal DVT in hospitalized patients [11]. Using the Padua score to classify VTE risk was shown to be suboptimal [12], with inferior ability to identify medical patients who were not critically ill for risk of VTE [13].

The Caprini score not only integrated the general condition, past medical history, and perioperative conditions of the patients but also included relevant preventive measures. Caprini RAM was originally developed in 1991, and updated in 2013, 2019 [14, 15]. The Caprini score has been validated in assessing the VTE risk of critically ill surgical patients, general surgery patients, and urologic surgery patients [14, 16]. However, there is still a lack of large-scale studies in orthopedics especially with primary joint replacement, to analyze the efficacy of the Caprini score in predicting VTE risk. Therefore, the purpose of this study was to evaluate the effectiveness of the Caprini RAM in primary THA/TKA patients during hospitalizations.

## Methods

### Patient population

The study population was extracted from the National Inpatient Sample of Healthcare Cost and Utilization Project (HCUP) in 2019 compiled by the Agency for Healthcare Research and Quality, which includes the largest collection of longitudinal hospital care data in the United States. The inclusion criteria were age ≥ 18 years and primary hip or knee arthroplasty, while exclusion criteria were arthroplasty revision and age < 18 years. Data were queried with procedure codes for primary THA/TKA International Classification of Diseases, Tenth Revision Clinical Modification (ICD − 10-CM). In 2019, a total of 229,134 patients out of 7,083,805 patients met inclusion and exclusion criteria.

### VTE determination

VTE defined as patients with DVT or PE was confirmed by duplex ultrasonography, venography, computed tomography pulmonary angiography or other methods. Deep vein thrombosis included acute thrombosis of lower-extremity veins including iliac, femoral, popliteal, or calf veins. A total of 1,077 patients out of 229,134 patients were diagnosed with VTE. The patients were divided into the VTE (*n* = 1,077) and non-VTE groups (*n* = 228,057).

### Caprini score

The Caprini score is calculated based on VTE risk factors according to Caprini RAM (2013 Version, Supplementary Table 1). Points are weighted according to their risk factors to calculate the Caprini score.

### Data analysis

Independent t-tests and Chi-square test were used to compare baseline conditions between VTE (both DVT and PE) and non-VTE group. The ability of caprini to identify VTE patients was evaluated by plotting ROC curves and calculating AUC values ​​through sensitivity and specificity. Integrate Bootstrap with the above analysis to enhance the reliability of the results. A logistic regression model was used to calculate the odds ratio for factors including Caprini RAM and presented in the form of a forest graph. All analyses were performed using statistical software R version 4.2 and a result was considered statistically significant at the *P* < 0.05 level of significance.

## Result

In our study, we analyzed a total of 229,134 patients who underwent primary joint replacement. Of these, 1,077 patients were included in the VTE group, while 228,057 patients were included in the non-VTE group. When comparing the VTE group with the non-VTE group, we found that the VTE group had a higher average age (*P* < 0.001) and a higher proportion of patients with a BMI ≥ 25 (*P* < 0.05). There are no significant differences between the two groups when it came to gender and BMI ≥ 40 (Table 1).

**Table 1:** Demographics. Abbreviations: BMI, body mass index (calculated as the weight in kilograms divided by height in meters squared)

Criteria	non-VTE		VTE	*P* Value
	*n*=228,057		*n*=1,077	
Age, years	68(57.2-78.8)		72(60.6-83.4)	<0.0001
BMI(Kg/m²)				
BMI≥25	58,151(25.50%)		243(22.56%)	0.022
BMI≥40	15,504(6.8%)		71(6.59%)	0.693
Gender, N(%)	F=137,050(60.09%)		F=665(61.75%)	0.270
	M=91,007(39.91%)		M=412(38.25%)	
THA	115,249		577	-
TKA	112,808		500	-

**Fig. 1:**
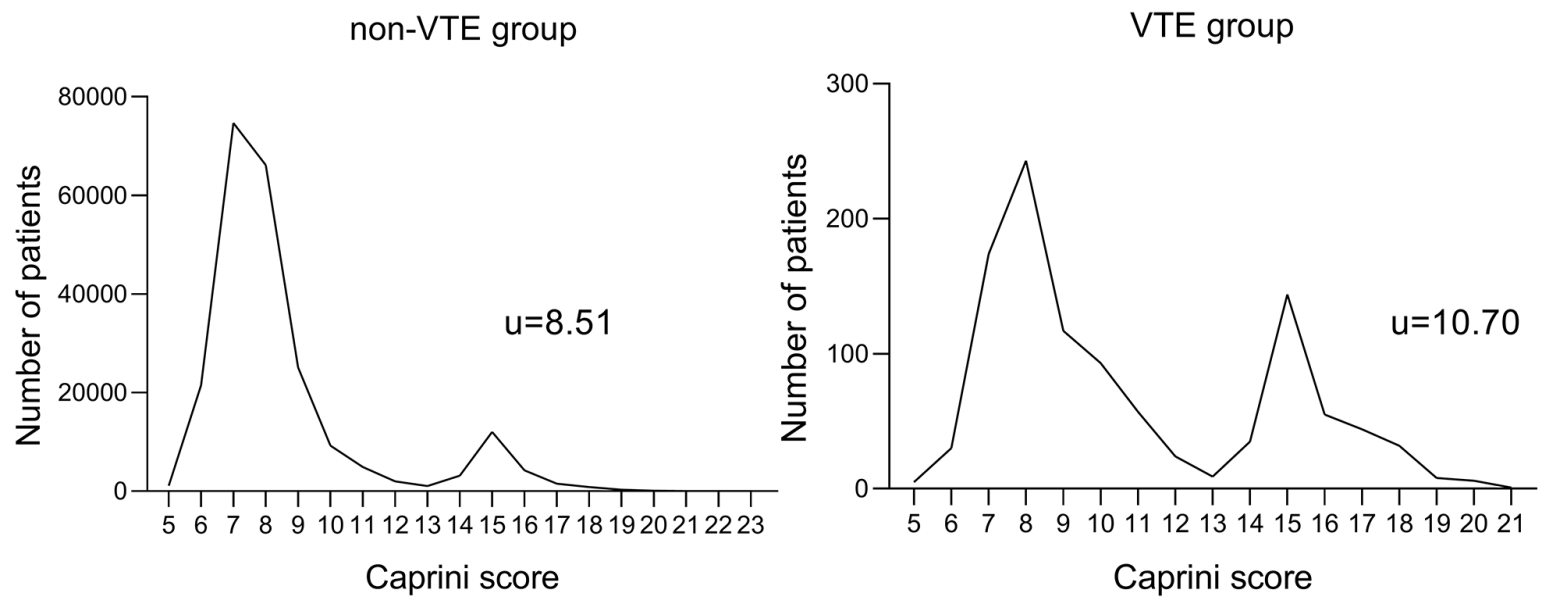
Describes the distribution of the population’s Caprini score

Two groups of patients’ Caprini scores were counted and drew their distribution as shown in Fig. 1, separately. The mean Caprini score was 10.70 in the VTE group, 8.51 in the non-VTE group, and a statistically significant difference was found between the two groups (*P* < 0.0001). There were two peaks observed in the distribution, with one peak at 7 in the non-VTE group and 8 in the VTE group, and another peak at 15 points in both groups (Fig. 1). The second peak at 15 points was mainly attributable to patients with hip fractures, who accounted for 30% of VTE patients in 10% of the total population (322 out of 1,077 VTE patients and 22,909 out of 229,134 population). The incidence of VTE was significantly higher in the hip fractures group (14‰) compared to the non-hip fractures group (3.7‰), and statistical analysis confirmed that the hip fractures group had a higher risk of thrombosis (*P* < 0.0001, Table 2).

**Table 2:** VTE events statistics for hip fractures and non-hip fractures groups

Criteria	non-hip fractrue	hip fracture	*P* Value
	*n*=206,225	*n*=22,909	
VTE Event	755	322	
VTE Rate	3.7‰	14‰	<0.0001

**Fig. 2:**
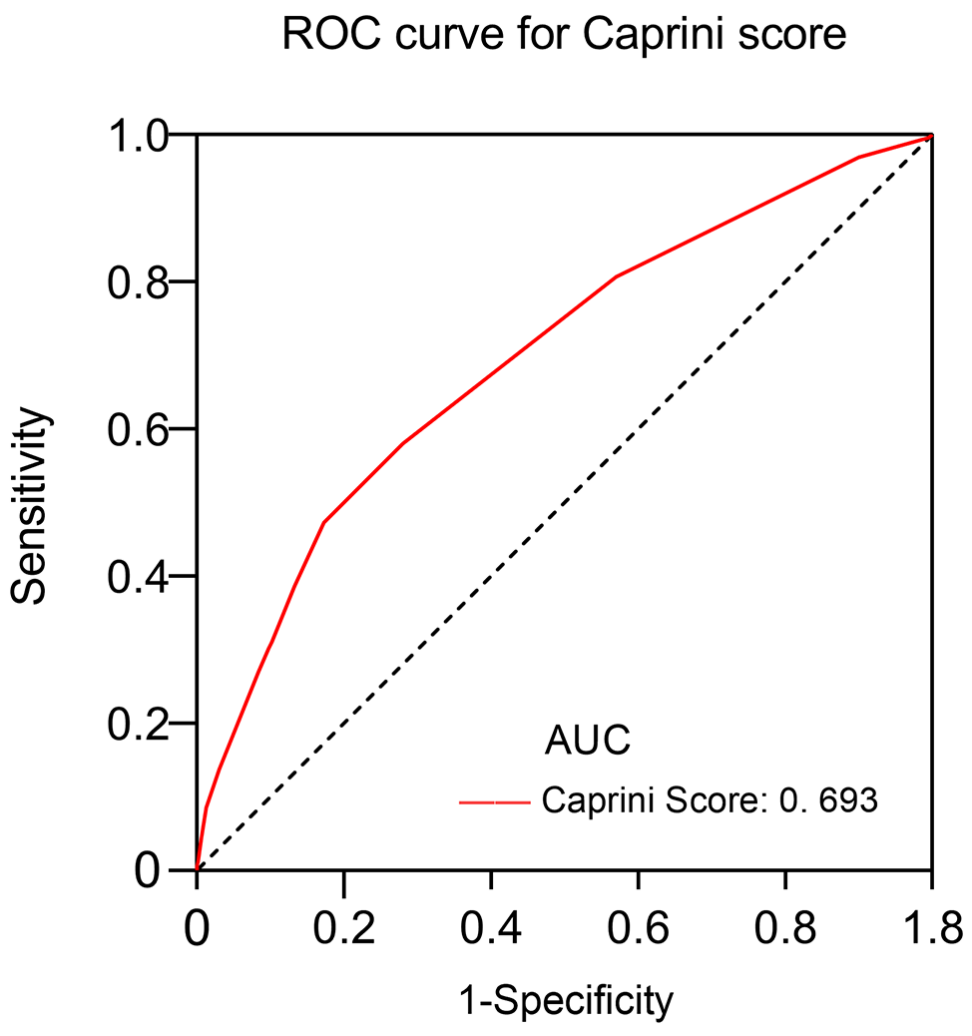
ROC curve analysis of Caprini score for the prediction of VTE. ROC, receiver operating characteristic curve; AUC, area under the curve

To further assess these findings, the ROC curve was drawn between the VTE and the non-VTE groups and calculating the area under the curve (AUC). According to ROC analysis (Fig. 2; Table 3), the optimal Caprini score cut-off value for predicting VTE was 9.5, with an AUC of 0.693 (95% CI, 0.677–0.710). This cut-off value had the highest Youden index (0.299), a sensitivity of 47.2% and a specificity of 82.7%.

**Table 3:** ROC curve of Caprini score-related evaluation parameters in the prediction of VTE.

	Caprini Score
Area under the curve	0.693
	(0.677-0.710)
Optimal threshold	9.5
Specificity	0.827
Sensitivity	0.472
Accuracy	0.826
Diagnose odds ratio	4.29
Positive predictive value	0.013
Negative predictive value	0.997

**Fig. 3:**
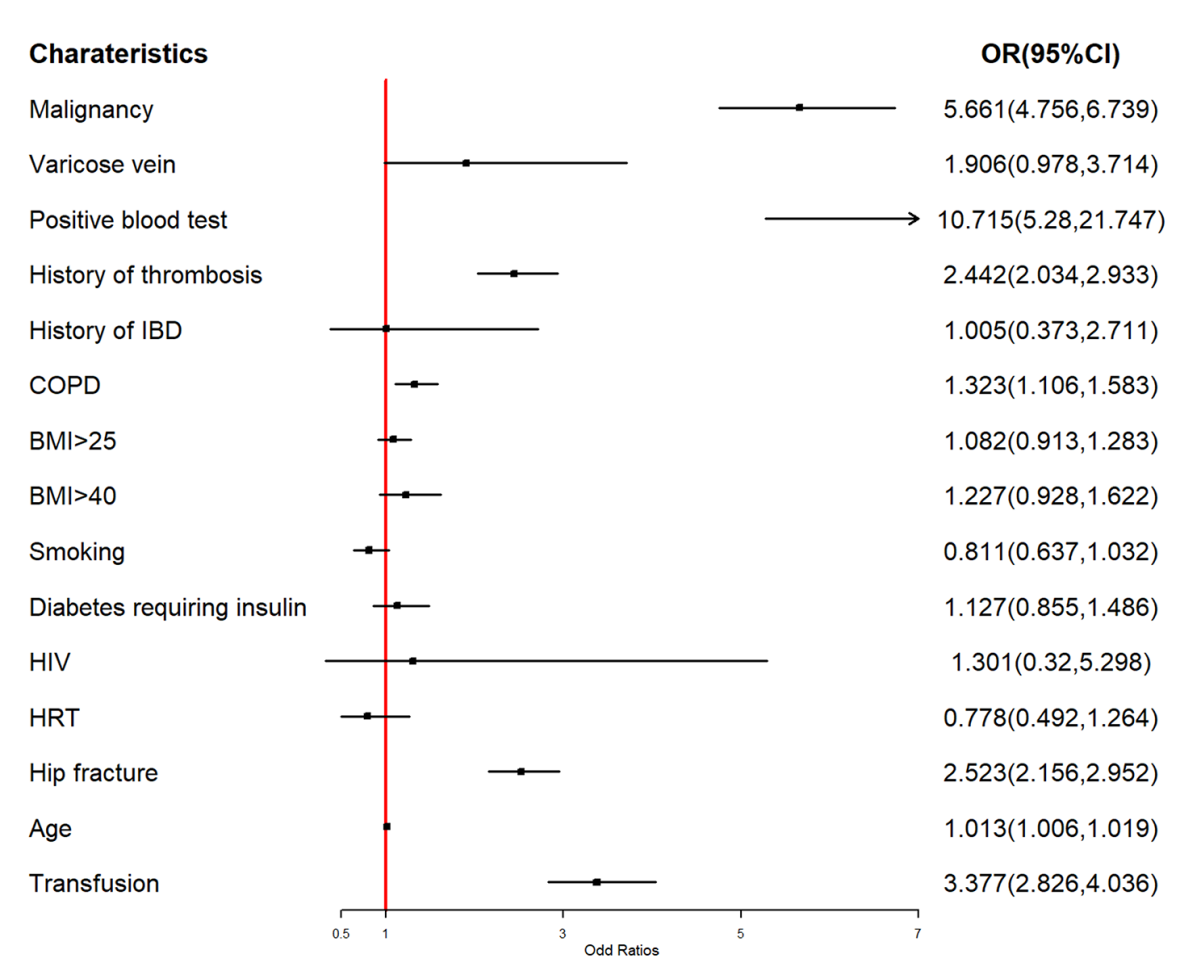
Multivariable logistic regression model showing the effect of Caprini indicators on VTE event after primary THA/TKA. Abbreviations: IBD, inflammatory bowel disease; COPD, chronic obstructive pulmonary disease; HIV, Human immunodeficiency virus; HRT, hormone replacement therapy

To elucidate the individual contributions of indicators including Caprini RAM versus VTE, multivariate logistic regression was used, and the results were plotted in a forest graph (Fig. 3). In this analysis, we found that several factors, including malignancy, varicose vein, positive blood test for thrombophilia, history of thrombosis, chronic obstructive pulmonary disease (COPD), hip fracture, transfusion, and age, were considered risk factors for VTE. Among these factors, positive blood test for thrombophilia (OR:10.715), malignancy (OR:5.661), and transfusion (OR:3.377) were found to be the three most important risk factors for VTE (Fig. 3).

**Fig. 4:**
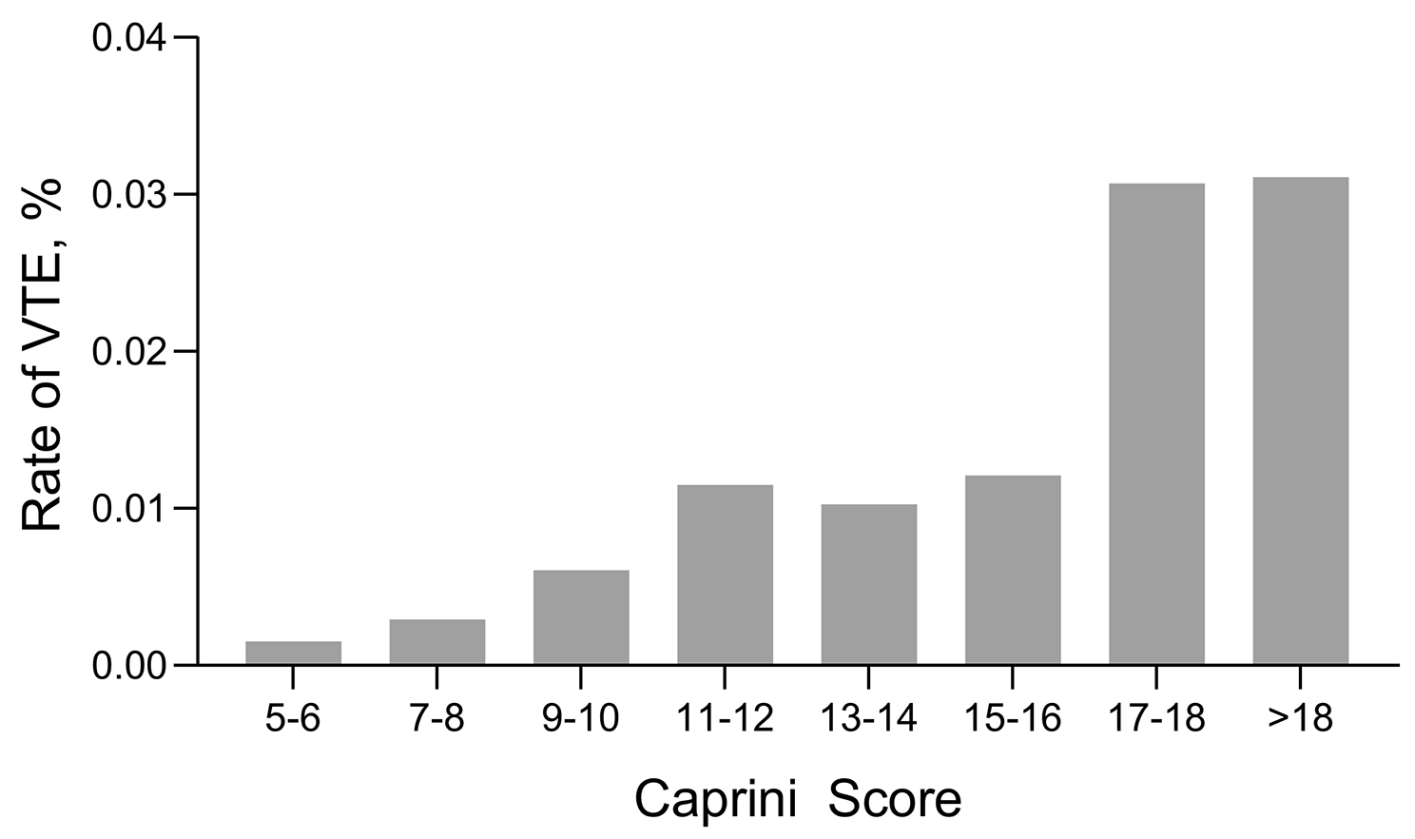
Caprini Scores and Venous Thromboembolism (VTE) Rates

Furthermore, we observed that the risk of thrombosis increased with an increase in the Caprini score (Fig. 4). A Caprini score greater than 16 was significantly associated with a higher risk of developing VTE events compared to a score of 15–16 (odds ratio [OR], 2.59; 95%CI, 1.98–3.38; *P* < 0.001). Similarly, a score of 11–12 was significantly associated with a higher risk compared to a score of 9–10 (odds ratio [OR], 1.91; 95%CI, 1.47–2.47; *P* < 0.001), and a score of 9–10 was associated with a higher risk compared to a score of 7–8 (odds ratio [OR], 2.06; 95%CI, 1.75–2.44; *P* < 0.001). Moreover, a score of 7–8 was significantly associated with a higher risk compared to a score of 5–6 (OR, 1.91; 95%CI, 1.36–2.70; *P* < 0.001) (Table 4, other odds ratios are also visible).

**Table 4:** Odds for venous thromboembolism stratified by Caprini score. CS: Caprini score, data are presented as odds ratios (95% CI), NS = no significant difference (*p* > 0.05)

	CS 7-8	CS 9-10	CS 11-12	CS 13-14	CS 15-16	CS 17-18	CS >18
CS 5-6	1.91(1.36-2.70)	3.95(2.76-5.65)	7.53(5.06-12.21)	6.71(4.30-10.48)	7.92(5.23-11.35)	20.49(13.70-30.65)	20.76(11.26-38.27)
*P* value	<0.001	<0.001	<0.001	<0.001	<0.001	<0.001	<0.001
CS 7-8		2.06(1.75-2.44)	3.94(3.10-5.00)	3.51(2.57-4.79)	4.14(3.49-4.90)	10.71(8.36-13.72)	10.85(6.43-18.30)
*P* value		<0.001	<0.001	<0.001	<0.001	<0.001	<0.001
CS 9-10			1.91(1.47-2.47)	1.70(1.23-2.36)	2.00(1.65-2.44)	5.19(3.98-6.76)	5.25(3.09-8.94)
*P* value			<0.001	<0.002	<0.001	<0.001	<0.001
CS 11-12				0.89(0.62-1.69)	1.05(0.81-1.36)	2.72(1.98-3.73)	2.76(1.58-4.82)
*P* value				**0.54(NS)**	**0.705(NS)**	<0.001	<0.001
CS 13-14					1.18(0.85-1.64)	3.05(2.10-4.44)	3.09(1.78-5.60)
*P* value					**0.322(NS)**	<0.001	<0.001
CS 15-16						2.59(1.98-3.38)	2.62(1.54-4.46)
*P* value						<0.001	<0.001
CS 17-18							1.01(0.58-1.78)
*P* value							**0.964(NS)**

## Discussion

It is estimated that 100,000 premature deaths are caused by VTE every year in the United States [6]. VTE has become one of the important complications of orthopedic surgery, especially in THA/TKA [9, 17]. Known factors that contribute to thrombosis, include venous stasis, vascular injury, and hypercoagulability [18]. In addition, hematological changes, tourniquet use, and reduced perioperative mobility resulting from THA and TKA surgery further increases the risk of VTE. The development of VTE can significantly increase the 30-day mortality rate in postoperative patients [19]. As a result, orthopedic surgeons are highly concerned about the risk of thrombosis in these patients [20]. While there are various measures available to prevent thrombosis, ranging from intermittent pneumatic compressive devices to pharmaceutical agents like low molecular weight heparin, aspirin, warfarin, and factor Xa inhibitors, an effective thrombosis risk assessment system is still lacking. Thromboprophylaxis is recommended for up to 35 days from the day of surgery rather than for only 10 to 14 days [9]. Symptoms associated with thrombosis formation may include lower limb pain and swelling, as well as chest pain and hemoptysis. Therefore, it is crucial to identify risk factors that can predict the likelihood of VTE.

To our knowledge, this is the largest study to focus on this topic. The results showed that the VTE group had significantly older age and higher BMI (Table 1), which is consistent with previous knowledge that older age and obesity are risk factors for thrombosis [13, 21]. The peak of Caprini score showed a bimodal distribution (Fig. 1), with the second peak caused by hip fracture leading to prolonged bed rest and venous stasis before THA. Additionally, patients with hip fractures have a 3.78-fold higher risk of VTE as compared to those without hip fractures (Table 2). It is obvious that hip fracture is one of the important risk factors for the development of VTE. Similarly, numerous studies have pointed out that hip fracture is an important risk factor for VTE [22, 23].

The ROC curves analysis revealed that the optimal cut-off value for the Caprini score in predicting VTE was 9.5, with an area under the curve (AUC) of 0.693 (Fig. 2; Table 3). This result is consistent with recently reported findings by Krauss et al [24]. Thus, patient with Caprini scores greater than 9.5 were classified as high-risk, whereas others were classified as low-risk. In our study, 66,871 (29.18%) were identified as high-risk groups. Thus, the Caprini RAM showed a certain predictive power with an AUC of 0.693. Although relatively lower sensitivity of 47.2%, high specificity of 82.7% appears to be more important for VTE prediction.

Although age contribution is limited (OR:1.013, Fig. 3), it is still considered a risk factor for VTE due to its age-cumulative effect. It cannot be ignored that some indicators were excluded, which seems to indicate that these indicators may not contribute to the VTE event. In this context, it is necessary to re-verify the effect of these indicators on promoting VTE and explore new indicators to enhance the risk assessment model in predicting VTE.

In the meantime, the study found that an increased Caprini score was associated with increased risk of VTE (Fig. 4; Table 4). Logistic regression results show that for every 2-point increase in the Caprini score within the range of 5–12, the risk approximately doubled. However, there was no significant increase in risk for scores between 11–12 and 13–16 (*P* > 0.05). Similar findings were observed for scores between 17–18 and greater than 18 points (Table 4), meaning patients within the same score range had similar VTE risk. Early in its inception a Caprini score greater than 5 was considered as high risk for general surgery patients [25]. Since total joint replacement alone adds 5 points to the score, this cutoff was not useful. Combined with these findings, we propose a new risk stratification for the Caprini score in primary THA/TKA patients: very low risk (5–6), low risk (7–8), intermediate risk (9–10), high-risk (11–16) and very high risk (> 16). Although the Caprini score may not be an ideal predictive model for VTE after primary THA/TKA, this new risk stratification enhances its usefulness. However, this study has several limitations. First, it was a retrospective study conducted in a single country and method of thromboprophylaxis was not mentioned. Second, the database does not provide information regarding the timing or other details about the occurrence of VTE events. Third, due to the diverse range of hospitals contributing data to the HCUP database, ensuring accuracy and consistency in ICD coding practices and variable identification across institutions poses a challenge. These factors may lead to potential bias in research findings. As a result, it is crucial to approach the conclusions of this study with caution, acknowledging the need for ongoing verification and refinement in future clinical research. Additionally, further research should involve multiple countries and cities and verify the effectiveness of the new risk stratification model under different VTE preventive measures.

## Conclusions

In summary, this study demonstrated that Caprini RAM has moderate predictive power for VTE risk in patients with primary THA/TKA. Nevertheless, some indicators within the Caprini score seem to have limited efficacy in predicting thrombosis. Therefore, it is crucial to identify novel methods or indicators that can accurately predict VTE events after primary THA/TKA. However, this new risk stratification proposed gives us a better understanding of the Caprini score, allowing us to more clearly stratify patients into different risk categories after THA/TKA. This makes it possible to utilize patient-specific VTE prevention measures and ultimately achieving the purpose of reducing the incidence of VTE.

### Electronic supplementary material

Below is the link to the electronic supplementary material.

Supplementary Material 1

Supplementary Material 2

## Data Availability

All other data can be obtained from the authors upon reasonable request.

## Abbreviations

HCUP: Healthcare Cost and Utilization Project
RAM: Risk assessment model
VTE: Venous Thromboembolism
THA: Total hip arthroplasty
TKA: Total knee arthroplasty
DVT: Deep vein thrombosis
PE: Pulmonary embolism
BMI: Body mass index
ROC: Receiver operating characteristic
AUC: Area under the ROC curve
ACCP: American College of Chest Physicians
AAOS: American Association of Orthopaedic Surgeons
ICD − 10-CM: International Classification of Diseases, Tenth Revision Clinical Modification
IRB: institutional review board
OR: Odds ratio
CS: Caprini score
IBD: Inflammatory bowel disease
COPD: Chronic obstructive pulmonary disease
HIV: Human immunodeficiency virus
HRT: Hormone replacement therapy
