# Supplementary material for: Utilization of the Caprini risk assessment model(RAM) to predict venous thromboembolism after primary hip and knee arthroplasty: an analysis of the Healthcare Cost and Utilization Project(HCUP)
Source: Thromb J. 2024 Jul 24;22:68. doi: 10.1186/s12959-024-00633-4 (PMC11267675; doi:10.1186/s12959-024-00633-4)
Supplement: Supplementary file 2 — Supplementary Material 2 [file 12959_2024_633_MOESM2_ESM.docx]

Positive blood test for thrombophilia, including Factor V Leiden/activated protein C resistance, antithrombin III deficiency, protein C & S deficiency, dysfibrinogenemia, 20,210 A prothrombin mutation, lupus anticoagulant, antiphospholipid antibodies, myeloproliferative disorders (including thrombocytosis), disorders of plasminogen and plasmin activation, heparin-induced thrombocytopenia, hyperviscosity syndromes, and homocysteinemia, HIV.
